# Long Term Marine Biodiversity Monitoring in Coastal Antarctica: Are Fewer Rare Species Recruiting?

**DOI:** 10.1111/gcb.70341

**Published:** 2025-07-08

**Authors:** David K. A. Barnes, Adriana Giles, Pati Glaz, Sean McLoughlin, Alice Clement, Simon A. Morley

**Affiliations:** ^1^ British Antarctic Survey NERC Cambridge UK

**Keywords:** marine recruitment, monitoring, rare species, settlement panels, Southern Ocean biodiversity

## Abstract

The physical environment of nearshore Southern Ocean (coastal Antarctica) is altering rapidly in response to climate change, but also has other long cyclicity due to El Nino Southern Oscillation and Southern Annular Mode. Detecting biological responses to such physical change, which is complex in time and space, is very challenging not least because of remoteness, difficulty of access, frequency of iceberg destruction and short funding cycles but also the paucity of research stations with SCUBA (or ROV) facilities. At one of those few, Rothera, Adelaide Island on the West Antarctic Peninsula, we immersed arrays of artificial substrata (settlement panels) for 1 year repeatedly for over two decades. Whilst many ‘mature assemblages’ are monitored at nearshore sites around the world, there are few of similar duration for recruitment and colonisation. We report the variability in annual biodiversity descriptive statistics with the crucial context of also recording adjacent long (here defined as > 1 decade) term seabed disturbance and biophysical oceanography at Rothera. We ask how variable is annual colonisation, recruitment and early community development in Antarctica's shallows, what aspect of recruitment changes over two decades and in what way? Of 40 recorded, most species recruiting to our panels at 12 m depth at Adelaide Island (67.568° S, 68.127° W) were rare, comprising cheilostome and cyclostome bryozoans, polychaetes, calcarea and demosponge sponges, hydroid cnidarians and ascidians. The most striking finding was a sustained decrease in total richness of recruits over time, mainly due to loss of rare species. Unlike losses of seasonal sea ice, iceberg disturbance and benthos mortality, such findings are unlikely to be climate‐forced responses. This raises important questions of whether this is a chance finding, (the data only spans 20 years), driven by a recent complex of stressors and most of all is losing rare species a wider polar problem?

## Graphical abstract

Barnes et al. report 21 years of monitoring marine invertebrate recruitment to artificial substrata (settlement panels) with contextual environmental variables at the West Antarctic Peninsula. Traditional measures of recruitment and colonisation, such as density and space occupation (% cover) varied but with no obvious temporal trend. Species richness and rare species strongly declined across the study period without clear environmental correlating drivers, showing the importance of robust baselines to future interpretation of complex ecological responses to global physical changes.

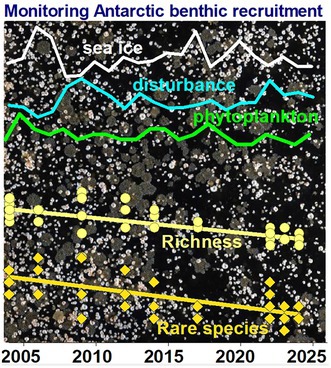

## 1 Introduction

Climate and ecosystems are dynamic across many scales, and thus how best to detect signals and trends of direct and indirect human impacts has long been an important goal. In the sea, artificial substrata have been one key way of monitoring colonisation, recruitment, early community development, and spatial competition from tropics to polar regions for more than half a century (Schuhmacher 1988). Such substrata particularly target encrusting (sessile) macro‐biota. This may provide earlier detection of non‐indigenous species establishment and spread, as well as responses to climate change and other stressors than, for example, in the slower rate of change in mature (less exposed to disturbance) assemblages (Ashton et al. 2017). The importance of ecology baselines and long‐term monitoring is often stressed but difficult to maintain funding for, so settlement panels are rarely monitored annually for > 5 years anywhere (but see Schuhmacher 1988 [20 years]; Dayton 1989 [10 years]; Schoener et al. 1978 [7 years]; Thomas 2009 [9 years]). However, it is also important to note that the biota fouling artificial substrata may not always reflect those on adjacent natural substrata (Dayton et al. 2016).

The seabed of the Southern Ocean remains the only place where virtually all of the fauna are native, most are endemic, and direct human impacts are very rare, making it one of the few places where recent measurements are truly a representative baseline. Change in the polar regions is amongst the most rapid, fundamental, and visible; for example, vast areas of white sea ice are turning to blue open water, with global consequences. The West Antarctic Peninsula (WAP) region has been a hotspot of physical change for some decades, with sustained seasonal sea ice losses, glacier retreat, and changes to the food web, for example, as shown by phytoplankton compositional and size change (Rogers et al. 2020). The physical environment of the nearshore Southern Ocean is not only showing rapid recent responses to climate change but also has other potential long cyclicity forced by El Niño Southern Oscillation, Southern Annular Mode, and stratospheric ozone losses (now recovering). As with Southern Ocean sea ice and temperature, change in assemblage and ecosystem is extremely complex, and detection of dynamic responses to recent anthropogenic stressors is hampered by so few measures of biodiversity on the seabed in time and space—exactly where most polar species (and nearly all endemics) live [see lists of species, taxon trees and their distributions www.SCARMarBIN.be].

Artificial substrata have been successfully used to characterise variability in % cover, density, richness at various taxonomic levels, and evenness at many global sites, but > 10 sites around each of the Arctic and Antarctic (Schoener et al. 1978; Dayton 1989; Bowden et al. 2006). Half a century ago it was realized there was important interannual as well as seasonal variability (Sutherland and Karlson 1977; Schoener et al. 1978) but it has proved difficult and rare to run artificial substrata or other marine colonisation experiments for more than 5 years. Even those few polar or long‐term studies have revealed fundamental properties of the biota there, such as massive supra‐annual variability in recruitment (Dayton 1989) and up to a doubling of growth with just 1°C warming (Ashton et al. 2017). We ask how variable is annual colonisation, recruitment, and early community development in Antarctica's shallows, and if we monitor this long term what aspect of recruitment changes and in what way?

## 2 Methods and Results

Between four and six high density perspex panels of size 14 cm × 14 cm were immersed and positioned on rocky substrata at 12 m depth at Adelaide Island, Antarctica (67.568° S, 68.127° W) using SCUBA diving. These were positioned next to large boulders in an attempt to offer some protection from iceberg scour. Such replicate panels were immersed across nine of the last 21 years up to 2024 (placed underwater for 12 months each beginning in the austral summer, for example of panel set up see Bowden et al. 2006). Panels were all emersed at the same site (South Cove, Ryder Bay) for the same period (1 year) at 1–5 year intervals between 2004 and 2024. All panels were immersed in the same down facing orientation, fixed to leave just 5 mm between the panel surface and the rocky substratum (thus excluding macro‐predators). At the end of each year that the 4–6 replicate panels were immersed, they were recovered for analysis, to give a sample size of 4–6 for each study year. All macroscopic life was identified to the highest taxonomic resolution from the central area of 10 cm × 10 cm of each panel by eye using high powered light microscopy (Leica M165c). Only biota in the central 10 cm × 10 cm area was recorded to minimise so‐called edge effects influenced by turbulent eddies at the periphery of the panel (following Bowden et al. 2006 and Ashton et al. 2017). The % cover was measured by covering all biota with an inelastic net marked out in mm^2^ so that the total number of mm^2^ occupied by each colonising species could be summed. Although these panel surfaces were very small areas relative to the size of the bay, they contained most of the encrusting species ever reported there, suggesting they are representative of the richness of the epifaunal assemblage. Here we report variability in annual biodiversity descriptive statistics of those panels in the context of seabed disturbance (iceberg scour frequency detailed in Barnes et al. 2024) and biophysical oceanography (oceanographic variables detailed in Venables et al. 2020) at the same location and timing. In relation to annual recruitment we show weekly mean sea temperature (°C) at 15 m depth, the number of days the sea surface was frozen into ‘fast ice’ (sea ice duration), the ice scour anomaly (compared with mean ice scour over the study period) and duration of phytoplankton abundance (how many months each year that the biomass of phytoplankton at 15 m depth exceeded 200 mg per m^3^). The anomaly of ice scour was calculated using the formula (the value of ice scour in a given year—mean ice scour across study years)/mean ice scour across study years.

We used non‐Metric Multidimensional Scaling (nMDS) as a method to better visualize (flatten) into two dimensions, patterns which are truly in more than two dimensions. Such an ordination of data was followed up with SIMPER (Similarity Percentage) analysis in PRIMER‐e software (v 7.0; Clarke and Gorley 2015). This method can be used to indicate the particular variables, or in the current study ‘species’, which are most influential to the differences observed between groups.

**FIGURE 1:**
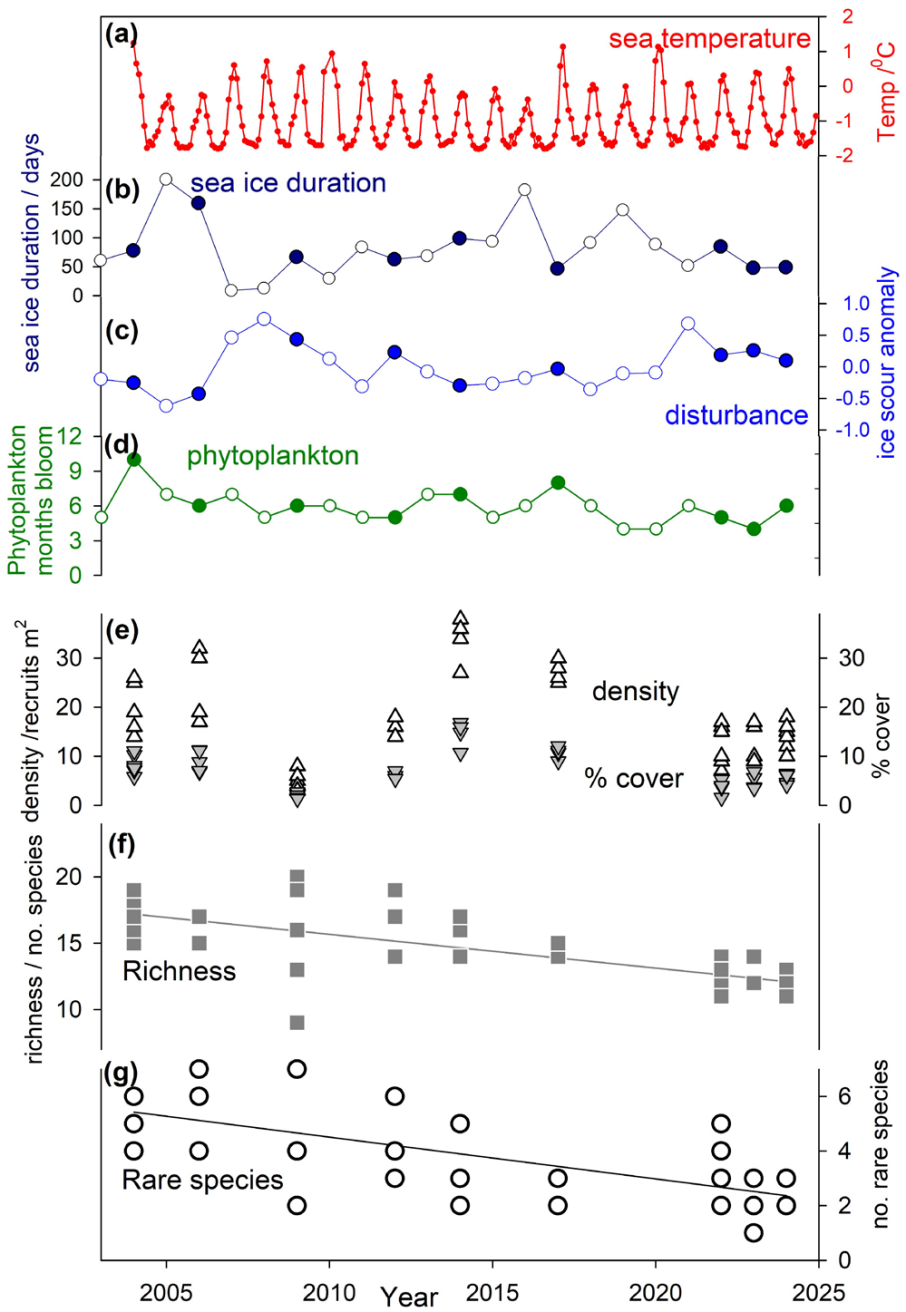
Antarctic environmental and marine recruitment to panels over 20 years. The environmental data shown are: Sea temperature (12 m, weekly mean), sea ice duration (days of fast ice per year), ice scour anomaly (relative to mean annual ice scour within the study period) and phytoplankton duration of abundance (months per year above 200 mg per m^3^). Filled circles indicate years in which panels were deployed whereas unfilled circles are years without panel deployments. Biological response data shown are: Space occupation (% cover; unfilled up‐pointing triangles), mean recruit density (per m^2^; grey filled down‐pointing triangles), species richness (number of species per panel, per year) and rare species presence (species with fewer than 1 per 1000 recruits).

Overall spirorbinid polychaetes (*Paralaeospira, Protolaeospira, Metalaeospira* and *Romanchella* genera) dominated recruit numbers over the study period (57%) but their proportion varied across years and was very low in the last 3 years (2022–2024) such that in 2023 and 2024, they were outnumbered by cheilostome bryozoans. Cheilostome bryozoans were by far the most speciose group (40% of all recruiting species) but many were rare (herein defined as < 1 in each 1000 recruits per year). The most abundant species on our panels reflected the more abundant species on natural surrounding (rocky) strata at the same depth, and likewise, the rare species present on panels were mainly those rare on rocks (see Barnes and Clarke 1998). Spatial coverage by colonists and recruit densities both decreased with increased ice scouring (ANOVA *r* ^2^ > 47%, *F* > 104, *p* < 0.01). Encrusting marine invertebrate recruitment density and cover could thus be linked to climate forcing (Barnes et al. 2024) but not time; they were similar at the start and end of 21‐year study (Figure 1).

**FIGURE 2:**
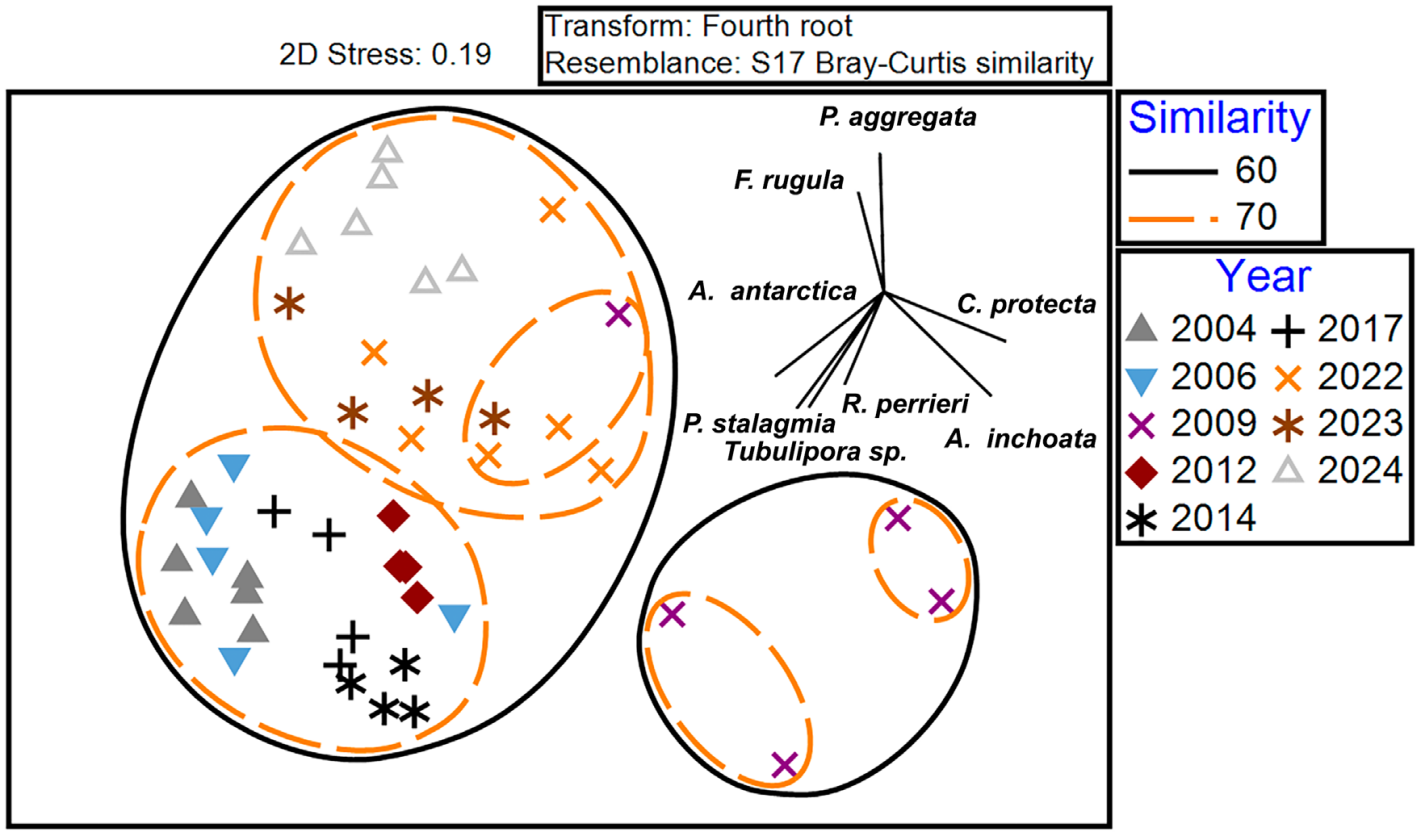
nMDS of the physical factors affecting encrusting assemblage structure on settlement panels. Assemblage data were fourth root transformed to reduce the influence of dominant species. Ellipses of 60%, 70%, and 80% similarity are shown. The vectors for the 8 most important species driving changes in assemblage structure (identified through SIMPER analysis) are indicated. Analysis conducted in PRIMER‐e v 7.0 (Clarke and Gorley 2015).

The proportion of recruits represented by spirorbinid polychaetes drastically decreased from ~80% to ~20%, but with a non‐linear pattern. There were clear differences in the assemblage structure between the earlier years (2004 to 2017, but with 2009 being anomalous) and later years (2022–2024; nMDS, 2D stress 0.19, Figure 2). Mean richness declined with time to 2024 (from 17 to 11.5 species per panel, see Figure 1, *r* ^2^ = 50.5, *F* = 42.8, *p* < 0.01), much of which was due to decreasing representation of rare species (from a maximum of 7–2.5 over two decades, *r* ^2^ = 40.3, *F* = 28, *p* < 0.01). Both richness and rarity were most closely correlated with the duration of the phytoplankton abundance/bloom (ANOVA *r* ^2^ > 25.4%, *F* > 17.9, *p* < 0.01). The number of months each year that seawater temperature was warmer than 0°C was strongly correlated with density, % cover, and richness (ANOVA *r* ^2^ > 4.1%, *F* > 11.4, *p* < 0.01). Significant factors influencing recruit density, % cover, richness, and rarity of assemblages on the settlement panels over the study period are shown in Table 1. Although multiple factors were significant influences, ice scour was most important to recruit density and % cover, whereas the duration of phytoplankton abundance (i.e., food for benthic suspension feeders) was most correlated to species richness and the presence of rare species (Table 1).

**TABLE 1:** Significant factors affecting density, % cover, richness, and rarity of assemblages growing on nearshore Antarctic settlement panels over 20 years. Results of multiple regression for the standardised factors (standardised values between +1.0 and −1.0), sea ice duration, ice scour, maximum summer sea temperature, duration (months) when seawater temperature, at which depth was > 0°C, and duration of the phytoplankton bloom (threshold months above 200 mg per m^3^). Backward elimination of terms was used until only significant terms were included (Minitab 2022). The acronyms are: DF (Degrees of freedom), Sequential sums of squares (Seq SS), Adjusted sums of squares (Adj SS), Adjusted mean square (Adj MS).

Factor	Source	DF	Seq SS	% Contribution	Adj SS	Adj MS	*F*‐value	*p*
Density	Regression	3	499.40	80.36	499.40	166.465	54.56	< 0.001
	Sea ice dur	1	59.97	9.65	144.37	144.371	47.32	< 0.001
	Ice scour	1	332.09	53.44	432.56	432.555	141.78	< 0.001
	Duration (months > 0C)	1	107.33	17.27	107.33	107.329	35.18	< 0.001
	Error	40	122.04	19.64	122.04	3.051		
	Lack‐of‐fit	5	31.09	5.00	31.09	6.218	2.39	0.057
	Pure error	35	90.95	14.64	90.95	2.599		
%Cover	Regression	3	2508.1	75.74	2508.1	836.04	41.62	< 0.001
	Sea ice dur	1	362.6	10.95	679.7	679.71	33.84	< 0.001
	Ice scour	1	1566.9	47.32	2094.7	2094.75	104.29	< 0.001
	Duration (months > 0C)	1	578.6	17.47	578.6	578.60	28.81	< 0.001
	Error	40	803.4	24.26	803.4	20.09		
	Lack‐of‐fit	5	194.4	5.87	194.4	38.89	2.23	0.073
	Pure error	35	609.0	18.39	609.0	17.40		
Richness	Regression	2	112.36	36.60	112.36	56.181	11.84	< 0.001
	Duration (months > 0C)	1	12.45	4.06	54.07	54.069	11.39	0.002
	Phytoplankton duration	1	99.91	32.55	99.91	99.911	21.05	< 0.001
	Error	41	194.61	63.40	194.61	4.747		
	Lack‐of‐fit	6	59.78	19.47	59.78	9.964	2.59	0.035
	Pure error	35	134.83	43.92	134.83	3.852		
Rarity	Regression	3	59.553	42.98	59.553	19.851	10.05	0.001
	Sea ice dur	1	22.729	16.41	48.403	48.403	24.51	0.001
	Ice scour	1	1.578	1.14	27.031	27.031	13.69	0.001
	Phytoplankton duration	1	35.247	25.44	35.247	35.247	17.85	0.001
	Error	40	78.992	57.02	78.992	1.975		
	Lack‐of‐fit	5	7.425	5.36	7.425	1.485	0.73	0.608
	Pure error	35	71.567	51.66	71.567	2.045		

## 3 Discussion

When coastal subtidal ecology is maintained, remarkable findings can be made that overturn fundamental understanding of systems (see Dayton 1989). Even within the wider region of West Antarctica, the WAP particularly has been characterised by rapid seasonal sea ice loss (Eayrs et al. 2021), which in turn has driven increased iceberg scouring of the coastal seabed (Barnes et al. 2024). Local and regional responses to retreat of Antarctic glaciers has been colonisation of fjords by benthic species, in other words increases in biomass, density and richness (see Zwerschke et al. 2022). In contrast the most striking and surprising finding of the current study was a sustained decrease in total richness of recruits over time (middle Figure 1). Most of the decrease of richness at Adelaide Island could be explained by a loss of rare species (lower plots of Figure 1). Unlike losses of seasonal sea ice, iceberg disturbance and benthos mortality (Barnes et al. 2024) such findings were a surprise and are not obviously climate‐forcing responses. As in many places around the world, rare (and small) species were an important part of the Antarctic assemblages. Most species designated rare in the current study were always rare, but some were more abundant than the 1 per 1000 recruits definition in some years, but not others. For example, one mainly rare species, *Chaperiopsis protecta*, constituted 10% of recruits in its most abundant year. How to analyse species which cross boundaries, for example, are sometimes rare and sometimes not, is a point that needs careful consideration. A key expectation of biodiversity response to multi stressors and climate change is ‘few winners, many losers’ and that has been predicted from modelled temperature change around Antarctica (Griffiths et al. 2017). Although we found exactly this situation with few species increases versus the decrease of many rarities, it is not clear if or how this is linked to any of the main known stressors.

The study site was approximately 6 km from the nearest retreating glacier terminus (Sheldon Cove), and it is possible that suspended inorganic particles (turbidity) have been an important influence, as it has been shown to impact particularly sessile and suspension‐feeding biota (Sicinski et al. 2012). Changes in phytoplankton composition and cell size have been amongst the most notable regional ecological responses to climate change (see Rogers et al. 2020) and such shifts could also potentially advantage some recruiting species over others. Both these potential explanations for richness, rarity, and macrofaunal compositional change can be climate‐forced and thus would be an indirect link to the study species. Again, longer time frames of observation should be valuable not just for detecting subtlety in assemblage‐level directional change but also for the drivers of it (e.g., climate change).

By some measures (recruit % cover, density and dominant species) there was little recent, directional change. It was notable that lows in % cover occurred following peaks of iceberg scouring (and low sea ice duration) and may have driven anomalous assemblage composition at times with varying time lags. Future studies, ideally with longer time series, may be able to elucidate more subtle and time‐lagged assemblage‐environment interactions. However, there has been massive change in other measures in the 20‐year study period (richness, rarity and polychaete numbers [from averages of > 600 per panel at the start of the study < 140 by the end, in Supporting Information]). Such trends of unexplained ‘background’ variability (i.e., not obviously linked to a known driver such as climate change, El Nino Southern Oscillation or Southern Annular Mode) are not reported much in the literature (Schuhmacher 1988; Schoener et al. 1978; Thomas 2009) and are comparable to or higher than found by manipulated warming (of similarly sized and replicated panels, see Ashton et al. 2017 [panels immersed for 9 months]). This sustained change is very different from rare one‐off pulses of recruitment found in the high Antarctic (Dayton 1989). Signals of temperature or phytoplankton change over the study period were minor (Venables et al. 2020) but were correlated with elements of assemblage structure, particularly richness and rarity (Table 1). Changes in the length of the feeding season and the duration of sea temperatures suitable for growth (for some species) are expected to alter competitive balance between species, potentially altering patterns of succession (Ashton et al. 2017; Barnes et al. 2021). As none of the panels in the current study were submerged for more than a year, it is possible that processes that develop over longer time frames (e.g., reproduction, predation, competition for food and space) could be underestimated. Multiyear studies are required for these, which can be challenging for polar funding and logistics support (research station roles are now restricted to ‘single winter’ [maximum ~16 months] in duration) as well as iceberg protection.

Seasonal sea ice has considerably reduced locally, regionally, and around both poles (Turner and Comiso 2017; Eayrs et al. 2021) which has typically led to increasing levels of iceberg scouring and consequent benthic mortality (e.g., in the Rothera shallows, see Barnes et al. 2024). The frequency of iceberg scouring has not been measured at many localities (Gutt and Starmans 2001; Deregibus et al. 2017). It is possible that if scouring levels measured locally are representative of wider patterns, then many populations of adults in the shallows have been depressed. This might be enough to hit the supply side of larvae (Underwood and Fairweather 1989), potential recruits of the rarer species (e.g., the bryozoans *Tubulipora* sp. and *Smittina rogickae* bryozoans) and in the case of demographic bottlenecks, rare variants (alleles and species) might be more likely to be lost. However, within the same period, some polychaete species (e.g., *Paralaeospira aggregata* ) increased, and others did not change (e.g., *Paralaeospira cavata*). Furthermore, recruitment of some of the most abundant species has also radically increased (e.g., the bryozoan *Fenestrulina rugula* Figure 2), as found in other encrusting species in some temperate nearshore locations (e.g., Ireland, see Thomas 2009). Other key species characteristic of earlier (2004–2017 assemblages) were *Antarctothoa antarctica* (bryozoan) and *Protolaeospira stalagmia* (polychaete), whereas the recovery from ice scour disturbance (in 2009) was more characterised by the bryozoans *Chaperiopsis protecta* and *Arachnopusia inchoata* (Table 1 and Figure 2).

Physical change around the polar oceans has quite a degree of uncertainty and can be highly complex with interactions between stressors (Gutt et al. 2015). Thus, signals of biodiversity or functional change in response to climate is likely to be even more difficult to tease out from ‘background noise’ (Rogers et al. 2020). Long‐term monitoring of biodiversity (e.g., in protected vs. non‐protected areas) is widely seen as essential to both combat a global current species loss crisis and quantify whether mitigation measures actually work. For biodiversity beyond charismatic mega‐fauna and krill in polar environments, a big question arises of how well do we know and understand natural variability over long time spans longer than ~5 years? The overall and unexplained losses of richness and rare marine invertebrate species we found in the current study are worrying, but are they reflective of wider areas or even just Adelaide Island over wider time spans? Most species in Antarctica are endemic and rare (see open access data in www.SCARMarBIN.be) so loss of local/regional species could often be losses of global biodiversity and yet nature in polar seas is so poorly monitored in space and time. The complexity and unexpected patterns described in the current work show not just how important longer term data sets are but also the cautiousness needed before accrediting short‐term change to stressors, such as climate‐forcing (even if the region is a hotspot of climate change as West Antarctica is).

## Author Contributions

**David K. A. Barnes:** conceptualization, data curation, formal analysis, investigation, methodology, writing – original draft, writing – review and editing. **Adriana Giles:** investigation, methodology, writing – review and editing. **Pati Glaz:** investigation, methodology, writing – review and editing. **Sean McLoughlin:** investigation, methodology, writing – review and editing. **Alice Clement:** investigation, methodology, writing – review and editing. **Simon A. Morley:** formal analysis, writing – original draft, writing – review and editing.

## Conflicts of Interest

The authors declare no conflicts of interest.

## Supporting information


Data S1

## Data Availability

The data that support the findings of this study are openly available in Dryad at https://doi.org/10.5061/dryad.tdz08kq9t. Biophysical oceanography data were obtained from NERC EDS UK Polar Data Centre at https://doi.org/10.5285/50ACB5B7‐5B42‐44CD‐A98E‐790BD367F204. Sea ice and iceberg scour frequency data were obtained from https://10.1016/j.cub.2024.03.036 (suppl https://www.cell.com/cms/10.1016/j.cub.2024.03.036/attachment/97f942b6‐7371‐41eb‐b6a6‐1616134c66b7/mmc1.pdf).

## References

[gcb70341-bib-0001] Ashton, G. V. , S. A. Morley , D. K. A. Barnes , M. S. Clark , and L. S. Peck . 2017. “Warming by 1°C Drives Species and Assemblage Level Responses in Antarctica's Marine Shallows.” Current Biology 27: 2698–2705. 10.1016/j.cub.2017.07.048. 28867203

[gcb70341-bib-0002] Barnes, D. K. A. , G. V. Ashton , S. A. Morley , and L. S. Peck . 2021. “1 Degrees C Warming Increases Spatial Competition Frequency and Complexity in Antarctic Marine Macrofauna.” Communications Biology 4, no. 1: 208. 10.1038/s42003-021-01742-w. 33594210 PMC7886862

[gcb70341-bib-0003] Barnes, D. K. A. , and A. Clarke . 1998. “The Biology of an Assemblage Dominant; the Encrusting Bryozoan *Fenestrulina* .” Invertebrate Biology 117, no. 33: 1–340.

[gcb70341-bib-0004] Barnes, D. K. A. , S. A. Morley , R. Mathews , A. Clement , and L. S. Peck . 2024. “Trajectory of Increased Iceberg Kill‐Off in West Antarctica's Shallows.” Current Biology 34: R488–R490. 38772332 10.1016/j.cub.2024.03.036

[gcb70341-bib-0005] Bowden, D. , A. Clarke , L. S. Peck , and D. K. A. Barnes . 2006. “Antarctic Sessile Marine Benthos: Colonisation and Growth on Artificial Substrata Over Three Years.” Marine Ecology Progress Series 316: 1–16.

[gcb70341-bib-0006] Clarke, K. R. , and R. N. Gorley . 2015. “Getting Started With PRIMER v7.” PRIMER‐e: Plymouth, Plymouth Marine Laboratory 20, no. 1: 1–296.

[gcb70341-bib-0007] Dayton, P. K. 1989. “Interdecedal Variation in an Antarctic Sponge and Its Predators From Oceanographic Climate Shifts.” Science 245: 1484–1486. 17776799 10.1126/science.245.4925.1484

[gcb70341-bib-0008] Dayton, P. K. , J. Shannon , K. Stacy , et al. 2016. “Surprising Episodic Recruitment and Growth of Antarctic Sponges: Implications for Ecological Resilience.” Journal of Experimental Marine Biology and Ecology 482: 38–55.

[gcb70341-bib-0009] Deregibus, D. , M. L. Quartino , K. Zacher , G. L. Campana , and D. K. A. Barnes . 2017. “Understanding the Link Between Sea Ice, Ice Scour and Antarctic Benthic Biodiversity – The Need for Cross‐Station and International Collaboration.” Polar Record 53, no. 2: 143–152. 10.1017/S0032247416000875.

[gcb70341-bib-0010] Eayrs, C. , X. Li , M. N. Raphael , and D. M. Holland . 2021. “Rapid Decline in Antarctic Sea Ice in Recent Years Hints at Future Change.” Nature Geoscience 14: 460–464.

[gcb70341-bib-0011] Griffiths, H. J. , A. J. S. Meijers , and T. J. Bracegirdle . 2017. “More Losers Than Winners in a Century of Future Southern Ocean Seafloor Warming.” Nature Climate Change 7: 749–754.

[gcb70341-bib-0012] Gutt, J. , N. Bertler , T. J. Bracegirdle , et al. 2015. “The Southern Ocean Ecosystem Under Multiple Climate Change Stresses‐An Integrated Circumpolar Assessment.” Global Change Biology 21, no. 4: 1434–1453. 10.1111/gcb.12794. 25369312

[gcb70341-bib-0013] Gutt, J. , and A. Starmans . 2001. “Quantification of Iceberg Impact and Benthic Recolonisation Patterns in the Weddell Sea (Antarctica).” Polar Biology 24: 615–619.

[gcb70341-bib-0014] Minitab . 2022. Minitab Statistical Software, Version 22. Minitab.

[gcb70341-bib-0015] Rogers, A. D. , B. A. V. Frinault , D. K. A. Barnes , et al. 2020. “Antarctic Futures: An Assessment of Climate‐Driven Changes on Ecosystem Structure, Function and Service Provision in the Southern Ocean.” Annual Review of Marine Science 12: 87–120. 10.1146/annurev-marine-010419-011028. 31337252

[gcb70341-bib-0016] Schoener, A. , E. R. Long , and J. R. DePalma . 1978. “Geographic Variation in Island Colonisation Curves.” Ecology 59: 367–382.

[gcb70341-bib-0017] Schuhmacher, H. 1988. “Development of Coral Communities on Artificial Reef Types Over 20 Years (Eilat, Red Sea).” In *Proceedings of 6th International Coral Reef Symposium*, 3, 379–384.

[gcb70341-bib-0018] Sicinski, J. , K. Pabis , K. Jazdzewski , A. Konopacka , and M. Blazewicz‐Paszkowycz . 2012. “Macrozoobenthos of Two Antarctic Glacial Coves: A Comparison With Non‐Disturbed Bottom Areas.” Polar Biology 35: 355–367.

[gcb70341-bib-0019] Sutherland, J. P. , and R. H. Karlson . 1977. “Development and Stability of the Fouling Community at Beaufort, North Carolina.” Ecological Monographs 47: 425–446. 10.2307/1942176.

[gcb70341-bib-0020] Thomas, R. 2009. “Larval Settlement and Benthic Recruitment: Variation Over Temporal and Spatial Scales.” PhD Thesis, University College Cork, Ireland.

[gcb70341-bib-0021] Turner, J. , and J. Comiso . 2017. “Solve Antarctica's Sea‐Ice Puzzle.” Nature 547: 275–277. 28726837 10.1038/547275a

[gcb70341-bib-0022] Underwood, A. J. , and P. G. Fairweather . 1989. “Supply‐Side Ecology and Benthic Marine Assemblages.” Trends in Ecology and Evolution 4: 16–20. 21227303 10.1016/0169-5347(89)90008-6

[gcb70341-bib-0023] Venables, H. J. , P. Holland , and M. P. Meredith . 2020. “Rothera Time Series.” https://www.bas.ac.uk/project/rats/.

[gcb70341-bib-0024] Zwerschke, N. , C. J. Sands , A. Roman‐Gonzalez , et al. 2022. “Quantification of Blue Carbon Pathways Contributing to Negative Feedback on Climate Change Following Glacier Retreat in West Antarctic Fjords.” Global Change Biology 28: 8–20. 10.1111/gcb.15898. 34658117

